# Fe_3_O_4_@SiO_2_@KIT-6@2-ATP@Cu^I^ as a catalyst for hydration of benzonitriles and reduction of nitroarenes

**DOI:** 10.1038/s41598-023-34409-z

**Published:** 2023-05-11

**Authors:** Zahra Moradi, Arash Ghorbani-Choghamarani

**Affiliations:** 1 Department of Chemistry, Faculty of Sciences, Ilam University, P.O. Box 69315516, Ilam, Iran; 2 Department of Organic Chemistry, Faculty of Chemistry, Bu-Ali Sina University, P.O. Box 6517838683, Hamedan, Iran

**Keywords:** Heterogeneous catalysis, Magnetic materials, Synthetic chemistry methodology

## Abstract

In this paper, a new type of magnetic mesoporous material (Fe_3_O_4_@SiO_2_@KIT-6@2-ATP@Cu^I^) was designed and synthesized and its application in the synthesis of amides and anilines was investigated. The structure of Fe_3_O_4_@SiO_2_@KIT-6@2-ATP@Cu^I^ was characterized and identified using FTIR, SEM, XRD, TGA, BET, VSM, and ICP techniques. An external magnet can easily remove the synthesized catalyst from the reaction medium, and be reused in several consequence runs.

## Introduction

Functional anilines are versatile intermediates for the preparation of agricultural chemicals, pigments, pharmaceuticals, and dyes^1–8^. Because of their importance, many methods have been developed for the reduction of nitroarenes to produce corresponding anilines. Generally, the methods can be classified into two types. In the common procedure, the stoichiometric reduction of the corresponding nitroarenes occurred using an appropriate reducing agent such as Na_2_S_2_O_4_, Fe, Sn, or Zn; this method often reasons environmental problems such as large amounts of waste acids and residues produced during the reaction. In the second procedure, the hydrogenation of nitro compounds is performed by metal catalysts in the presence of an appropriate catalyst^9–11^.

Amides are important raw materials for the production of detergents, lubricants, drug stabilizers, and mediators in peptide and protein synthesis^12–18^. For preparing amides from nitriles different methods have been reported in the literature, hydration of nitriles to the corresponding amides is one of the extensively studied procedures^19–24^.

Today, the use of magnetic nanoparticles (MNPs) in catalytic reactions is wildly studied. Magnetic mesoporous silica (MMS) nanoparticles due to their many important properties such as excellent stability (thermal and chemical), high surface area, simple and easy separation from the reaction medium, and recyclability, show excellent catalytic performance in a wide range of organic reactions^25–28^. In this research project, we have synthesized a new and efficient catalyst (Fe_3_O_4_@SiO_2_@KIT-6@2-ATP@Cu^I^) that has the advantage of both magnetic nanoparticles and mesoporous materials. In this research, the catalytic aspects of Fe_3_O_4_@SiO_2_@KIT-6@2-ATP@Cu^I^ have been examined for hydrating nitriles and reducing nitroarenes.

## Result and discussion

### Preparation and characterization of Fe_3_O_4_@SiO_2_@KIT-6@2-ATP@Cu^I^

**Figure 1:**
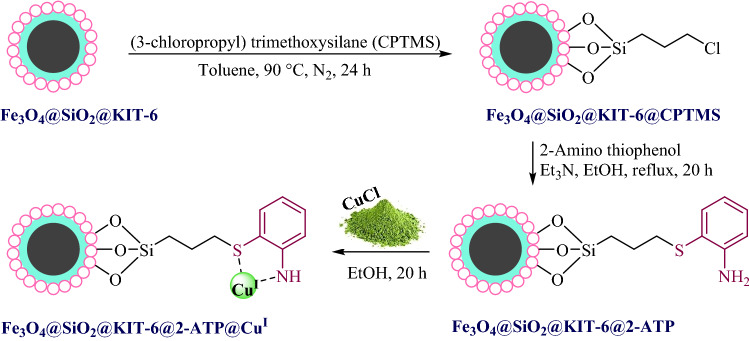
Stepwise preparation of Fe_3_O_4_@SiO_2_@KIT-6@2-ATP@Cu^I^.

The Fe_3_O_4_@SiO_2_@KIT-6 was prepared as mentioned procedure in our newly published work^29^. Subsequently, the prepared nanoparticles were first functionalized by (3-chloropropyl) trimethoxysilane and then reacted with 2-amino thiophenol. Finally, Cu(I) was coordinated with Fe_3_O_4_@SiO_2_@KIT-6@2-ATP (Fig. 1).

After designing and fabricating Fe_3_O_4_@SiO_2_@KIT-6@2-ATP@Cu^I^, the synthesized magnetic mesoporous structure was characterized by different techniques.

**Figure 2:**
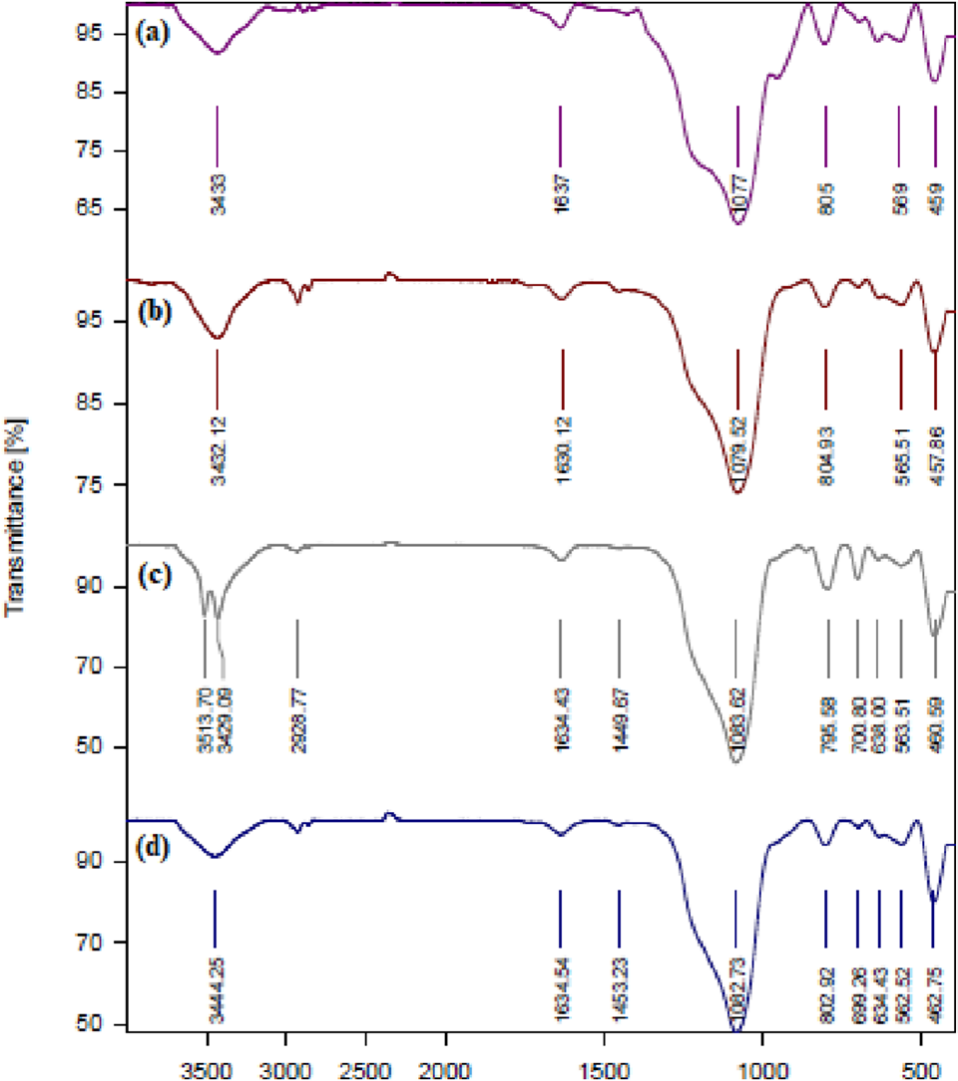
Infrared spectra of Fe_3_O_4_@SiO_2_@KIT-6 (**a**), Fe_3_O_4_@SiO_2_@KIT-6@CPTMS (**b**), Fe_3_O_4_@SiO_2_@KIT-6@2-ATP (**c**), Fe_3_O_4_@SiO_2_@KIT-6@2-ATP@Cu^I^ (**d**).

Infrared spectroscopy is one of the most widely used analyses for the identification of different functional groups of organic compounds. Various devices have been developed for infrared spectroscopy, the most widely used of which are Fourier transform devices. Therefore, Fourier transforms infrared spectroscopy (FT-IR) was used to identify the synthesized catalyst^30^. In Fig. 2, the synthetic steps of magnetic mesoporous catalyst have been studied by FT-IR analysis. Peaks appearing in 459 cm^−1^, 457 cm^−1^, 462 cm^−1^, 460 cm^−1^, 640 cm^−1^, 635 cm^–1^, and 634 cm^−1^ in the spectra of Fe_3_O_4_@SiO_2_@KIT-6 (Fig. 2a), Fe_3_O_4_@SiO_2_@KIT-6@CPTMS (Fig. 2b), Fe_3_O_4_@SiO_2_@KIT-6@2-ATP (Fig. 2c) and Fe_3_O_4_@SiO_2_@KIT-6@2-ATP@Cu^I^ (Fig. 2d) is related to the stretching vibration of the Fe–O bond. Also, the stretching vibration of the Si–O–Si bond in the region of 1077–1083 cm^−1^ appears in Fig. 2a–d. In the Fe_3_O_4_@SiO_2_@KIT-6@2-ATP spectrum (Fig. 2c), the peak is shown in 3513 cm^−1^ and 3429 cm^−1^ corresponding to the NH stretching vibration. In the spectrum, Fe_3_O_4_@SiO_2_@KIT-6@2-ATP@Cu^I^ (Fig. 2d) the peak that appears at 3444 cm^−1^ is belong to the N–H stretching vibration.

Scanning electron microscope (SEM) is one of the most common tools used in nanotechnology to analyze the morphology of nanostructural materials. The bombardment of the sample causes electrons to be released from the sample towards the positively charged plate, where these electrons become signals. The movement of the beam on the sample provides a set of signals based on which the microscope can display an image of the sample surface on the computer screen. So, in general, it is possible to obtain information including topography, components, and morphology of the sample^31^.

**Figure 3:**
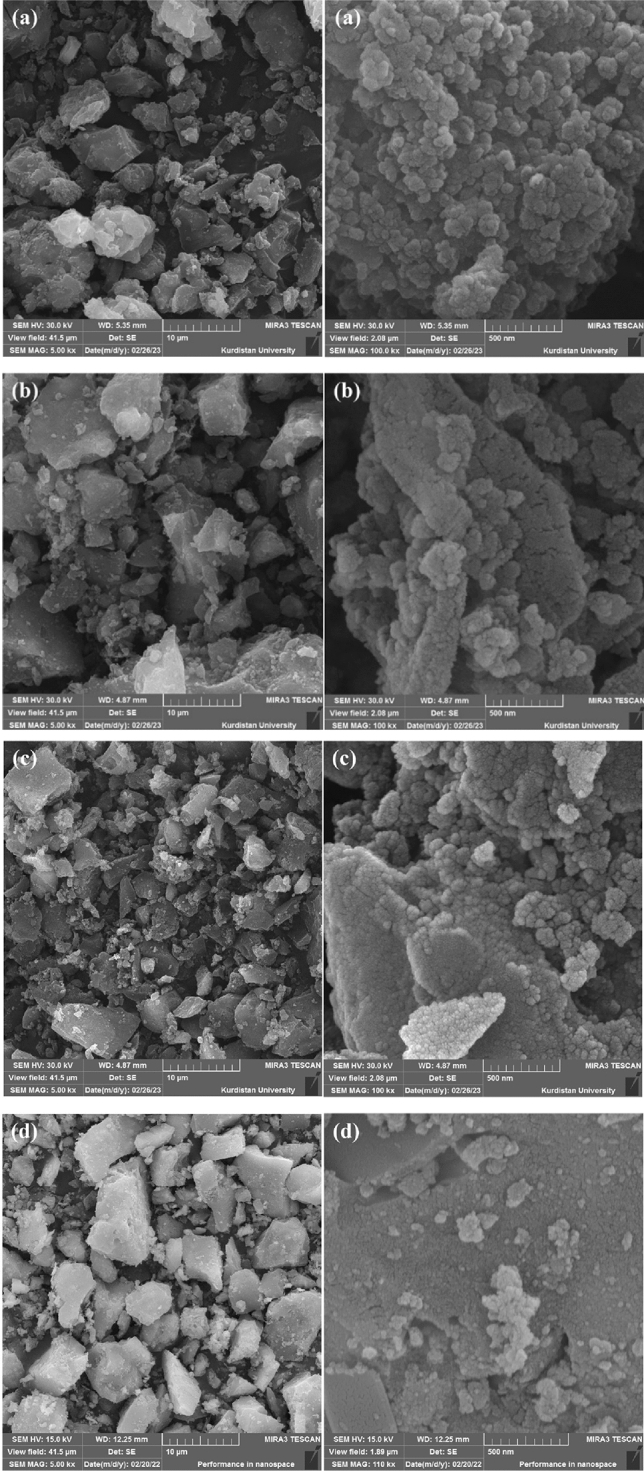
SEM image of Fe_3_O_4_@SiO_2_@KIT-6 (**a**), Fe_3_O_4_@SiO_2_@KIT-6@CPTMS (**b**), Fe_3_O_4_@SiO_2_@KIT-6@2-ATP (**c**), Fe_3_O_4_@SiO_2_@KIT-6@2-ATP@Cu^I^ (**d**).

To consider the morphology and particle shape of the magnetic mesoporous catalyst, the SEM image of Fe_3_O_4_@SiO_2_@KIT-6 (a), Fe_3_O_4_@SiO_2_@KIT-6@CPTMS (b), Fe_3_O_4_@SiO_2_@KIT-6@2-ATP (c), Fe_3_O_4_@SiO_2_@KIT-6@2-ATP@Cu^I^ (d) has been prepared, which has been brought in Fig. 3. The SEM images confirm the mesoporous catalyst formed in spherical shapes.

Thermogravimetric analysis (TGA), using a specific heating program and under a controlled atmosphere, measures weight changes in air or an inert atmosphere and records the mass reduction as a function of increasing temperature. Based on the results of thermal gravimetric analysis, it is possible to calculate the amount of combustible or vaporizable materials, including water and organic materials of the sample^32^.

**Figure 4:**
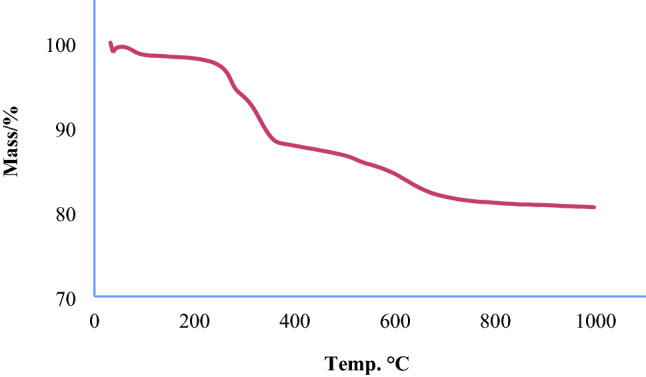
TGA diagram of Fe_3_O_4_@SiO_2_@KIT-6@2-ATP@Cu^I^.

Figure 4 shows the TGA diagram of a catalyst activated with 2-amino thiophenol. According to the diagram, the first weight loss (under 250 °C, about 3%) is related to the evaporation of adsorbed solvents. The second weight loss, which is about 12% and occurred at temperatures between 250 to 650 °C, is related to the removal of immobilized organic compounds, indicating that 2-amino thiophenol was successfully immobilized into KIT-6 magnetic channels.

**Figure 5:**
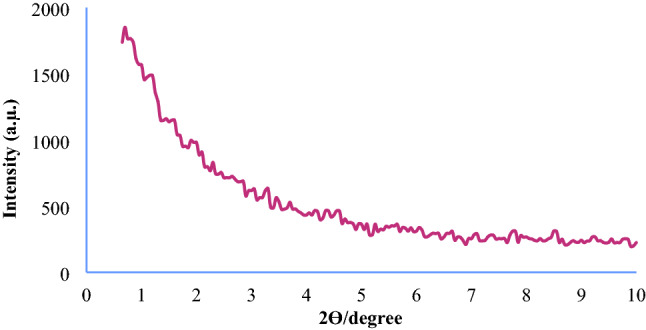
XRD patterns of Fe_3_O_4_@SiO_2_@KIT-6@2-ATP@Cu^I^ (low angle XRD).

**Figure 6:**
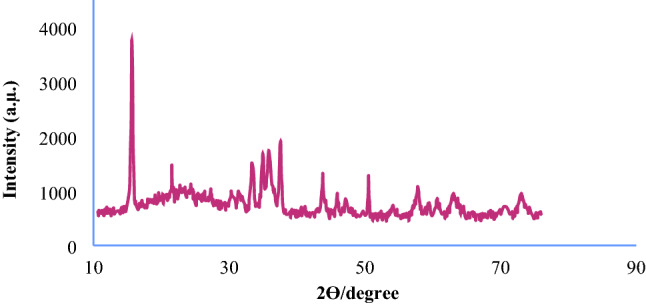
XRD patterns of Fe_3_O_4_@SiO_2_@KIT-6@2-ATP@Cu^I^ (wide angle XRD).

The X-ray diffraction pattern for the Fe_3_O_4_@SiO_2_@KIT-6@2-ATP@Cu^I^ catalyst is shown in Figs. 5 and 6, (low and wide angle respectively). The low-angle XRD spectrum shows in Fig. 5. In the high-angle XRD spectrum (Fig. 6**)**, the peaks appearing at 43.79°, 50.54°, and 73.24° correspond to the copper metal-stabilized into the channels of the catalyst, and a broad peak of 20–30 is related to the amorph silica layer^33,34^.

**Figure 7:**
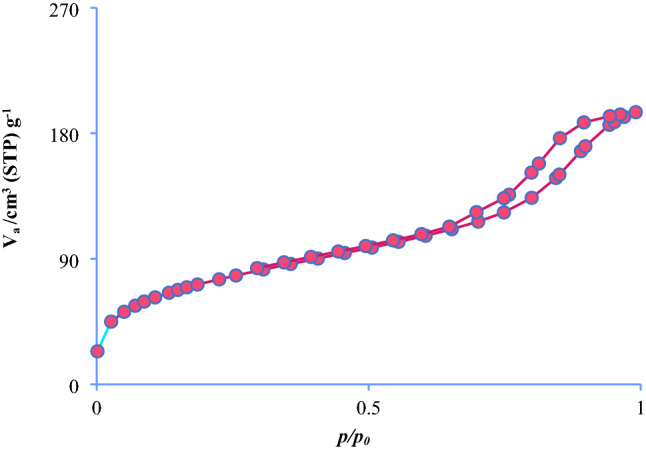
N_2_ adsorption–desorption isotherm of Fe_3_O_4_@SiO_2_@KIT-6@2-ATP@Cu^I^.

Figure 7 shows the nitrogen adsorption/desorption isotherm of Fe_3_O_4_@SiO_2_@KIT-6@2-ATP@Cu^I^. The isothermal adsorption–desorption curve for Fe_3_O_4_@SiO_2_@KIT-6@2-ATP@Cu^I^ shows type IV of IUPAC isotherms, indicating the magnetic material form in a mesoporous structure. The N_2_ adsorption–desorption isotherm had a sharp bend at P/P_0_, indicating capillary density in uniform mesopores.

The pore and surface properties of Fe_3_O_4_@SiO_2_@KIT-6@2-ATP@Cu^I^ were calculated and considered using a Brunauer-Emmet-Teller (BET) and Barret-Joyner-Halenda (BJH) methods (Tables 1 and 2).

**Table 1:** Nitrogen adsorption–desorption data was calculated by the BET method, for mesoporous magnetic catalyst (Fe_3_O_4_@SiO_2_@KIT-6@2-ATP@Cu^I^).

Vm	58.016 cm^3^g^−1^
a_s, BET_	252.51 m^2^g^−1^
Total pore volume(p/p = 0.990)	0.3016 cm^3^g^−1^
Mean pore diameter	4.78 nm

**Table 2:** BJH*-*plot data for Fe_3_O_4_@SiO_2_@KIT-6@2-ATP@Cu^I^.

V_p_	0.2491 cm^3^g^−1^
r_p_, peak(area)	1.22 nm
a_p_	157.52 m^2^g^−1^

**Figure 8:**
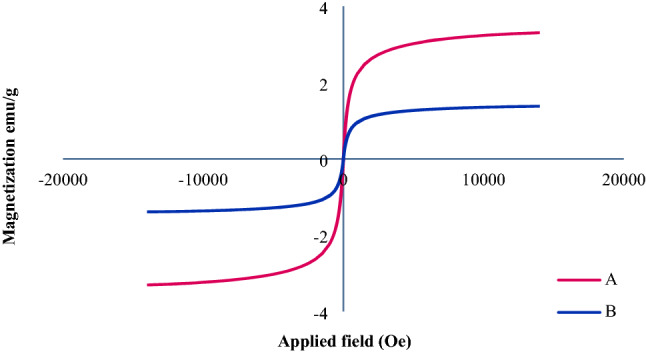
The magnetic curve of Fe_3_O_4_@SiO_2_@KIT-6 (**A**) and Fe_3_O_4_@SiO_2_@KIT-6@2-ATP@Cu^I^ (**B**).

As shown in Fig. 8, the magnetic property of Fe_3_O_4_@SiO_2_@KIT-6@2-ATP@Cu^I^ (1.38 emu/g) shows a significant decrease compared to Fe_3_O_4_@SiO_2_@KIT-6 nanoparticles (3.30 emu/g). The magnetic property of the mesoporous catalyst reflects the fact that the surface of the nanoparticles is coated with SiO_2_ and organic groups.

ICP analysis was used to determine the exact amount of loaded Cu on the magnetic mesoporous catalyst and showed a value of 1.11 × 10^–3^ mol/g.

### Catalytic studies

#### Hydration of benzonitriles to amides

After the structure characterized the magnetic mesoporous catalyst, its catalytic activity in the synthesis of amides was investigated. The reaction of 4-chlorobenzonitrile as model substrate was investigated in the presence of potassium hydroxide, various solvents such as water, ethanol, methanol, tetrahydrofuran, and 1-propanol, variable amounts of catalyst, and different temperature conditions. In protic polar solvents, coordination between the solvent and benzonitrile with the catalyst activates the cyano group in the nitrile substrate. Among protic polar solvents, 1-propanol led to more amide formation due to its coordination with the substrate^33,35^. Finally, 1-propanol, 40 mg of catalyst, 70 °C temperature, and 2 mmol of potassium hydroxide were selected as optimal conditions (Table 3).

**Table 3:** Optimization of conditions for the synthesis of amides in the presence of Fe_3_O_4_@SiO_2_@KIT-6@2-ATP@Cu^I^ catalyst.

Entry	Solvent	Catalyst (mg)	KOH (mmol)	Temperature (°C)	Time (min)	Yield (%) ^a^
1	EtOH	40	2	70	120	46
2	MeOH	40	2	Reflux	120	61
3	H_2_O	40	2	70	120	59
4	1-Propanol	40	2	70	120	94
5	THF	40	2	Reflux	120	70
6	1-Propanol	40	3	70	120	89
7	1-Propanol	40	4	70	120	87
8	1-Propanol	30	2	70	120	84
9	1-Propanol	20	2	70	120	79
10	1-Propanol	40	2	80	120	94
11	1-Propanol	40	2	60	120	83

^a^Isolated yield.

**Figure 9:**
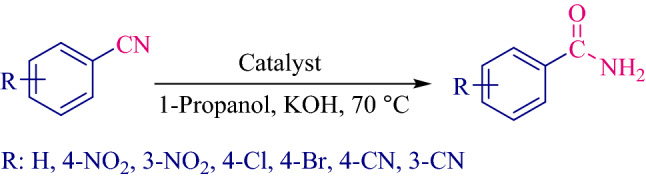
Hydration of benzonitriles to amides.

After obtaining the reaction conditions, the reaction of different benzonitriles was performed under optimal conditions and a variety of amides were synthesized (Fig. 9). The results including reaction times and yields are reported in Table 4**.**

**Table 4:** Synthesis of amides from benzonitriles in the presence of Fe_3_O_4_@SiO_2_@KIT-6@2-ATP@Cu^I^ catalyst.

Entry	Benzonitrile	Product	Time (min)	Yield (%)	Melting point (°C)
1			120	94	121–124^16^
2			120	90	199–200^36^
3			120	91	142–143^36^
4			120	94	171–173^15^
5			120	90	135–137^15^
6			120	91	190–192^16^
7			50	87	180–182^36^
8			52	88	224–225^36^

Reaction conditions: 4-Chlorobenzonitrile (1 mmol), potassium hydroxide (2 mmol), 1-Propanol, temperature, catalyst 40 mg.

**Figure 10:**
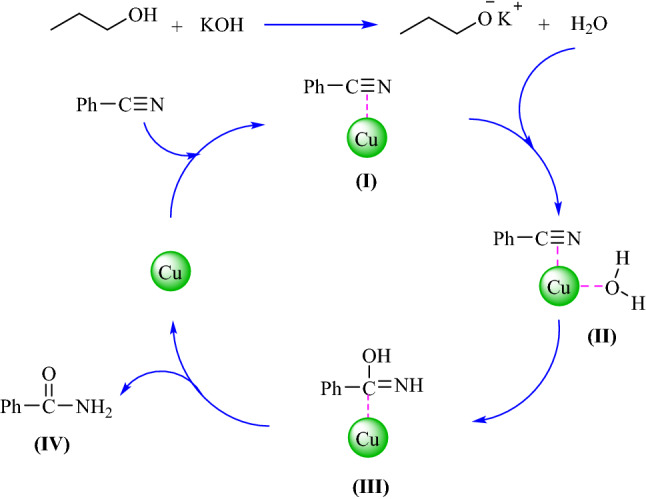
The suggested mechanism of amide synthesis in the presence of Fe_3_O_4_@SiO_2_@KIT-6@2-ATP@Cu^I^ as a catalyst.

The hydration mechanism in the presence of Fe_3_O_4_@SiO_2_@KIT-6@2-ATP@Cu^I^ is proposed in Fig. 10. Initially, the coordination of benzonitrile with the copper atom from the catalyst may lead to an increase in the electrophilicity of the nitrile carbon (intermediate I), which by the addition of HO^-^ ion leads to producing intermediate (III). Finally, the tautomerism of coordinated imines leads to an amide (IV)^36^.

### Reduction of nitroarenes to anilines

In another catalytic study, the activity of Fe_3_O_4_@SiO_2_@KIT-6@2-ATP@Cu^I^ in the reduction of nitroarenes to aromatic amines was investigated (Table 5). After considering the effect of different solvents, temperature conditions, and different amounts of catalyst; water as the solvent, and room temperature were selected as the optimal conditions for the preparation of anilines from nitroarenes.

**Table 5:** Optimization of reaction conditions in the presence of Fe_3_O_4_@SiO_2_@KIT-6@2-ATP@Cu^I^ catalyst.

Entry	Solvent	Catalyst (mg)	NaBH_4_ (mmol)	Time (min)	Yield (%)^a^
1	H_2_O	10	5	40	82
2	H_2_O	15	5	40	84
3	H_2_O	20	5	40	92
4	EtOH	20	5	40	81
5	H_2_O:EtOH	20	5	40	79
6	H_2_O	20	4	40	83
7	H_2_O	20	3	40	75
8	CH_3_CN	20	5	40	31
9	MeOH	20	5	40	49

^a^Isolated yield.

The effect of solvent on the reduction of nitroarenes was analyzed through articles and the results show that protic polar solvents are more suitable solvents for the reduction of nitroarenes than aprotic polar solvents^5^.

**Figure 11:**
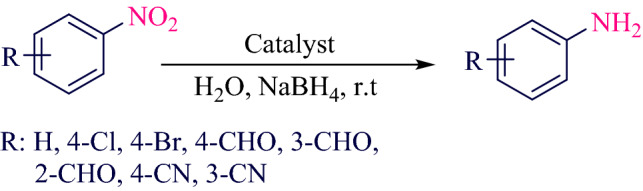
Reduction of nitroarenes to anilines.

After obtaining the optimal conditions, the reduction of different derivatives of nitroarenes to aromatic amines was performed and the results can be seen in Table 6 (Fig. 11).

**Table 6:** Synthesis of anilines from the reduction of nitroarenes Fe_3_O_4_@SiO_2_@KIT-6@2-ATP@Cu^I^ catalyst.

Entry	Nitro arene	Product	Time (min)	Yield (%)^a^	Melting point (°C)
1			60	89	Oil^3^
2			40	92	68–70^33^
3			35	90	64–65^41^
4			32	90	85–88^41^
5			32	90	85–86^41^
6			35	90	79–81^41^
7			20	92	79–80^42^
8			28	91	52–53^42^

Reaction conditions: 4-Chlorobenzene (1 mmol), NaBH_4_ (5 mmol), room temperature, catalyst 20 mg.

^a^Isolated yield.

**Figure 12:**
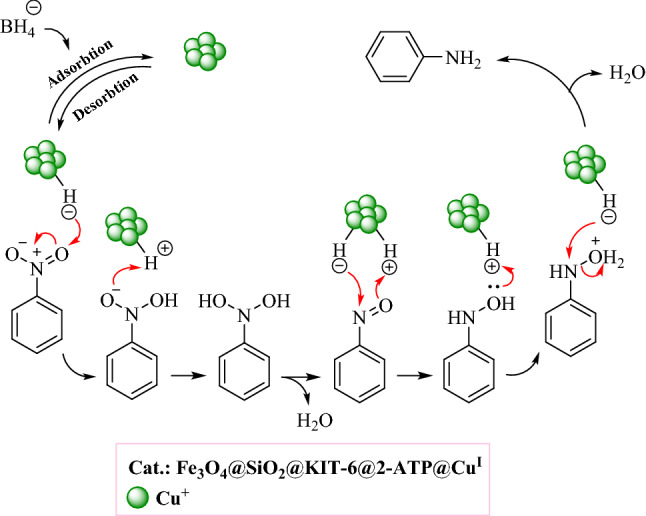
A suggested mechanism for the reduction of nitro compounds by NaBH_4_ in the presence of a catalytic amount of Fe_3_O_4_@SiO_2_@KIT-6@2-ATP@Cu^I^.

A proposed mechanism for the reduction of nitro compounds in the presence of Fe_3_O_4_@SiO_2_@KIT-6@2-ATP@Cu^I^ is provided in Fig. 12^37^.

### Reusability of the catalyst

**Figure 13:**
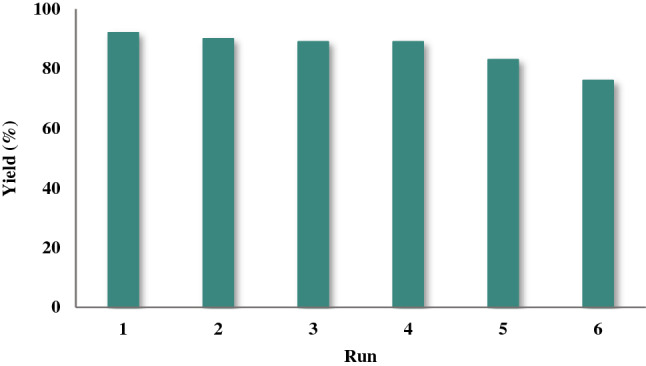
Catalyst recovery study.

To investigate the recovery of described catalyst, the reduction reaction of 1-chloro-4-nitrobenzene was selected as the sample reaction. The reaction was selected using 1-chloro-4-nitrobenzene, sodium borohydride, and water as the solvent in the presence of Fe_3_O_4_@SiO_2_@KIT-6@2-ATP@Cu^I^. After the reaction was complete, it was separated by an external magnetic field, washed with ethanol and water, and then used in the next run. This cycle was repeated up to four times (Fig. 13).

## Conclusions

In this paper, the Fe_3_O_4_@SiO_2_@KIT-6@2-ATP@Cu^I^ as a magnetic mesoporous catalyst was designed and synthesized through the combination of Fe_3_O_4_ and KIT-6 nanoparticles. The catalytic ability of this mesoporous magnetic material was studied for the preparation of benzamides and anilines. The reported procedure in this research project offers the advantages of reasonable yields and green reaction medium, versatile catalyst preparation procedure, short reaction times of catalytic reactions, easy separation (it could be easily separated and recovered due to its magnetic properties), catalyst recyclability, and high catalyst chemical stability.

In Table 7, the Fe_3_O_4_@SiO_2_@KIT-6@2-ATP@Cu^I^ magnetic catalyst was compared with other previously reported catalysts for the reduction of nitrobenzene. As is evidenced in this table, the obtained results by the described catalyst in this research are comparable with other reductive systems.

**Table 7:** Comparison of Fe_3_O_4_@SiO_2_@Kit-6@2-ATP@Cu^I^ with other catalysts for the reduction of nitrobenzene.

Entry	Catalyst	Conditions	Time (h)	Yield (%)^a^	Ref
1	SB‐Pd@MNPs	H_2_O, r.t, NaBH_4_	0.03	92	^43^
2	Pd/PPh_3_@FDU-12	EtOH, 40 °C, 10 bar H_2_	1	99	^29^
3	SiO_2_@APTES@β-CD@Pd-PDR	H_2_O, r.t, NaBH_4_	3	99	^44^
4	Co–N/C-800	EtOH, 100 °C, H_2_	4	96	^45^
5	[Co(κS,N-tfmp2S)_3_]	MeOH, 40 °C, MeNHNH_2_	4	92	^46^
6	Fe_3_O_4_@SiO_2_@KIT-6@ 2-ATP@Cu^I^	H_2_O, r.t, NaBH_4_	1	89	This work

^a^Isolated yield.

## Experimental

### Synthesis of Fe_3_O_4_@SiO_2_@KIT-6@2-ATP

Fe_3_O_4_@SiO_2_@KIT-6 nanoparticles were synthesized using a method previously reported in the literature^38^. In a 50 mL balloon, Fe_3_O_4_@SiO_2_@KIT-6 (1 g) was sonicated for 30 min in toluene (25 mL), then, 1.5 mL of (3-chloropropyl) trimethoxysilane (CPTMS) was added and the resulting mixture was stirred for 24 h at 90 °C under nitrogen atmosphere. The obtained solid was washed with dichloromethane (50 mL) and dried in an oven. In a round bottom balloon, a mixture of Fe_3_O_4_@SiO_2_@KIT-6@CPTMS (1 g), and one gram of 2-amino thiophenol (2-ATP) was refluxed in the presence of triethylamine (2 mL) in ethanol for 20 h. After separating the obtained precipitate and washing it with ethanol, it was dried at 50 °C^39^.

### Synthesis of Fe_3_O_4_@SiO_2_@KIT-6@2-ATP@Cu^I^

The solid precipitate that was obtained in the previous step, was dissolved in ethanol and 2 mmol of CuCl was added and refluxed for 20 h. After the end of the reaction, the Fe_3_O_4_@SiO_2_@KIT-6@2-ATP@Cu^I^ catalyst was separated and washed several times with ethanol^40^.

### The general method for the hydration of benzonitriles to amides

To perform hydration of benzonitrile, in a 5 mL round bottom flask, benzonitrile (1 mmol), potassium hydroxide (2 mmol), and 40 mg of Fe_3_O_4_@SiO_2_@KIT-6@2-ATP@Cu^I^ were added to 1-propanol and the mixture was stirred at 70 °C. The progress of the reaction was followed by TLC. After the completion reaction, the catalyst was separated by an external magnet and the corresponding product was extracted.

#### 4-Cyanobenzamide

^1^HNMR (300 MHz, DMSO-*d6*) δ 8.01 (2H, d, J = 8 Hz), 7.95 (2H, d, J = 8 Hz), 7.66 (s, 2H).

#### 4-Nitrobenzamide

^1^HNMR (300 MHz, DMSO-*d*6) δ 8.28 (2H, d, J = 8.1), 8.08 (2H, d, J = 8.1), 7.71 (s, 2H).

### The general method for the reduction of nitroarenes to anilines

To prepare anilines from nitroarenes, a mixture of nitroarene, NaBH_4_ (5 mmol), and 20 mg catalyst was stirred at room temperature. TLC was used to monitor the progress of the reaction and the product was obtained in high yield after catalyst isolation.

#### 4-Bromoaniline

^1^HNMR (300 MHz, CDCl_3_): δ 7.23 (2H, d, J = 7 Hz), 6.57 (2H, d, J = 7 Hz), 3.53 (s, 2H).

#### 2-Aminobenzyl alcohol

^1^HNMR (300 MHz, CDCl_3_): δ 6.93–7.05 (m, 2H), 6.49–6.62 (m, 2H), 4.95 (s, 1H), 4.87 (s, 2H), 4.38 (s, 2 H) (Supplementary Information).

## Supplementary Information

Supplementary Information.

## Data Availability

All data generated or analyzed during this study are included in this published article [and its supplementary information files].
