# Supplementary material for: Fe3O4@SiO2@KIT-6@2-ATP@CuI as a catalyst for hydration of benzonitriles and reduction of nitroarenes
Source: Sci Rep. 2023 May 11;13:7645. doi: 10.1038/s41598-023-34409-z (PMC10175259; doi:10.1038/s41598-023-34409-z)
Supplement: Supplementary file 1 — Supplementary Information. [file 41598_2023_34409_MOESM1_ESM.pdf]

$Fe_3O_4@SiO_2@KIT$-6@2-ATP@Cu^I as a catalyst for hydration of benzonitriles and reduction of nitroarenes

Zahra Moradi, Arash Ghorbani-Choghamarani *

A) 4-Cyanobenzamide (Figure1):

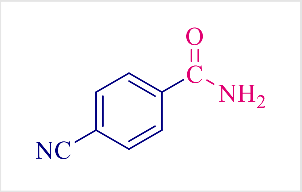

4-Cyanobenzamide: ^{1}HNMR (300 MHz, DMSO-*d6*) δ 8.01 (2H, d, J= 8 Hz), 7.95 (2H, d, J= 8 Hz), 7.66 (s, 2H).

B) 4-Nitrobenzamide (Figure 2):

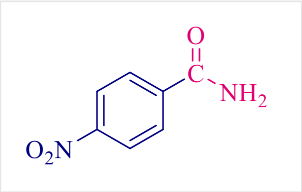

4-Nitrobenzamide: ^{1}HNMR (300 MHz, DMSO-*d*6) δ 8.28 (2H, d, J= 8.1), 8.08 (2H, d, J= 8.1), 7.71 (s, 2H).

C) 4-Bromoaniline (Figure 3):

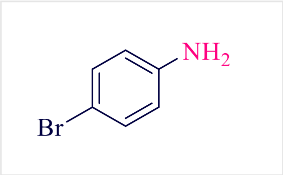

4-Bromoaniline: ^{1}HNMR (300 MHz, $CDCl_3$): δ 7.23 (2H, d, J= 7 Hz), 6.57 (2H, d, J= 7 Hz), 3.53 (s, 2H).

D) 2-Aminobenzyl alcohol (Figure 4):

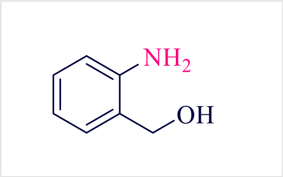

2-Aminobenzyl alcohol: ^{1}HNMR (300 MHz, $CDCl_3$): δ 6.93- 7.05 (m, 2H), 6.49- 6.62 (m, 2H), 4.95 (s, 1H), 4.87 (s, 2H), 4.38 (s, 2 H).

^{1}HNMR:

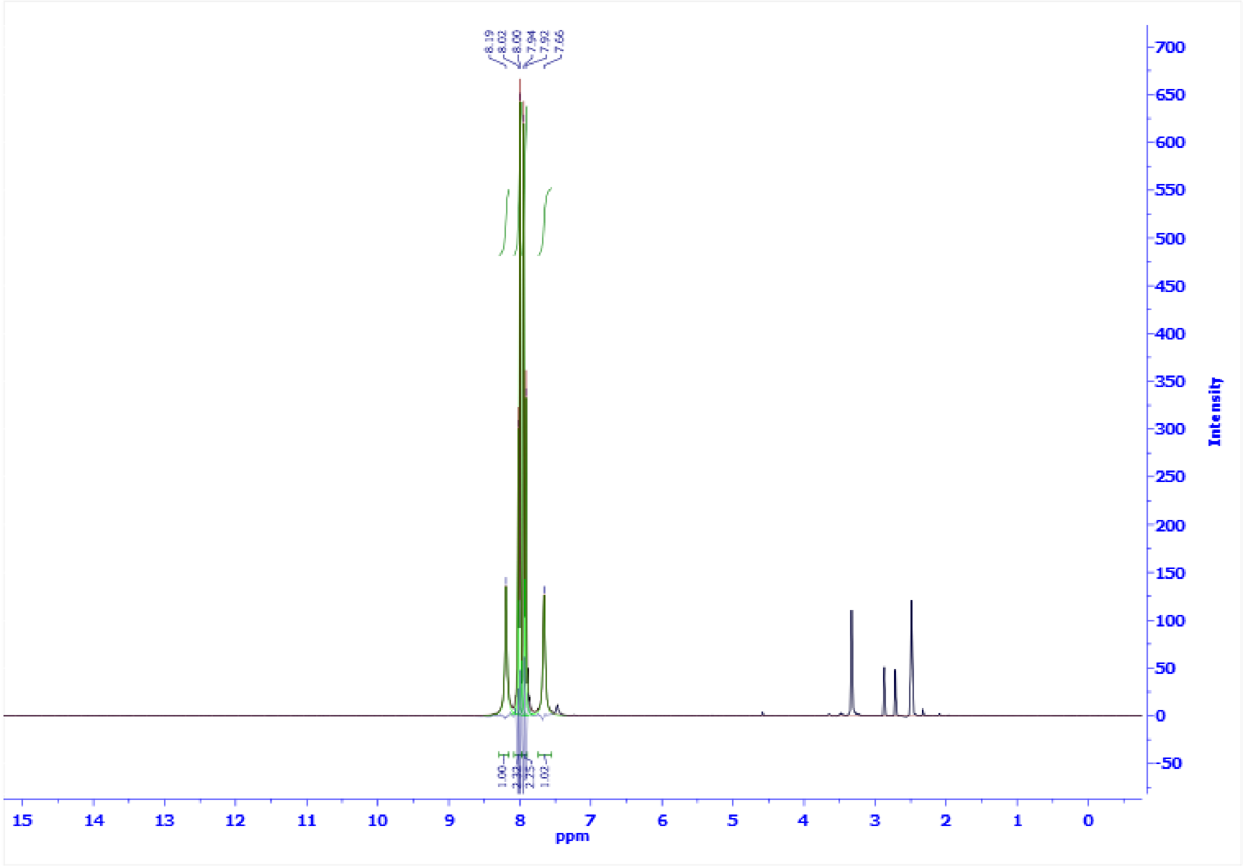

Figure 1. The ^{1}H NMR A

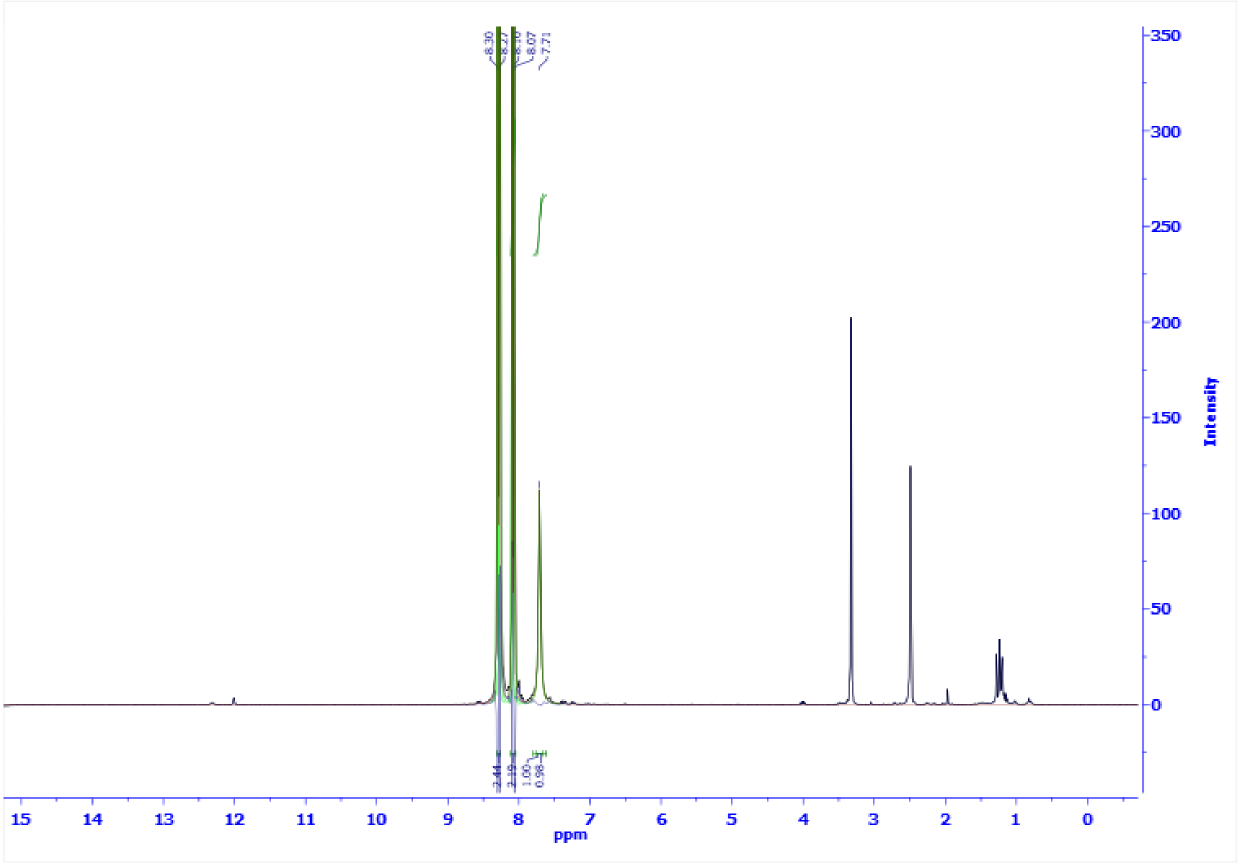

Figure 2. The ^{1}H NMR B

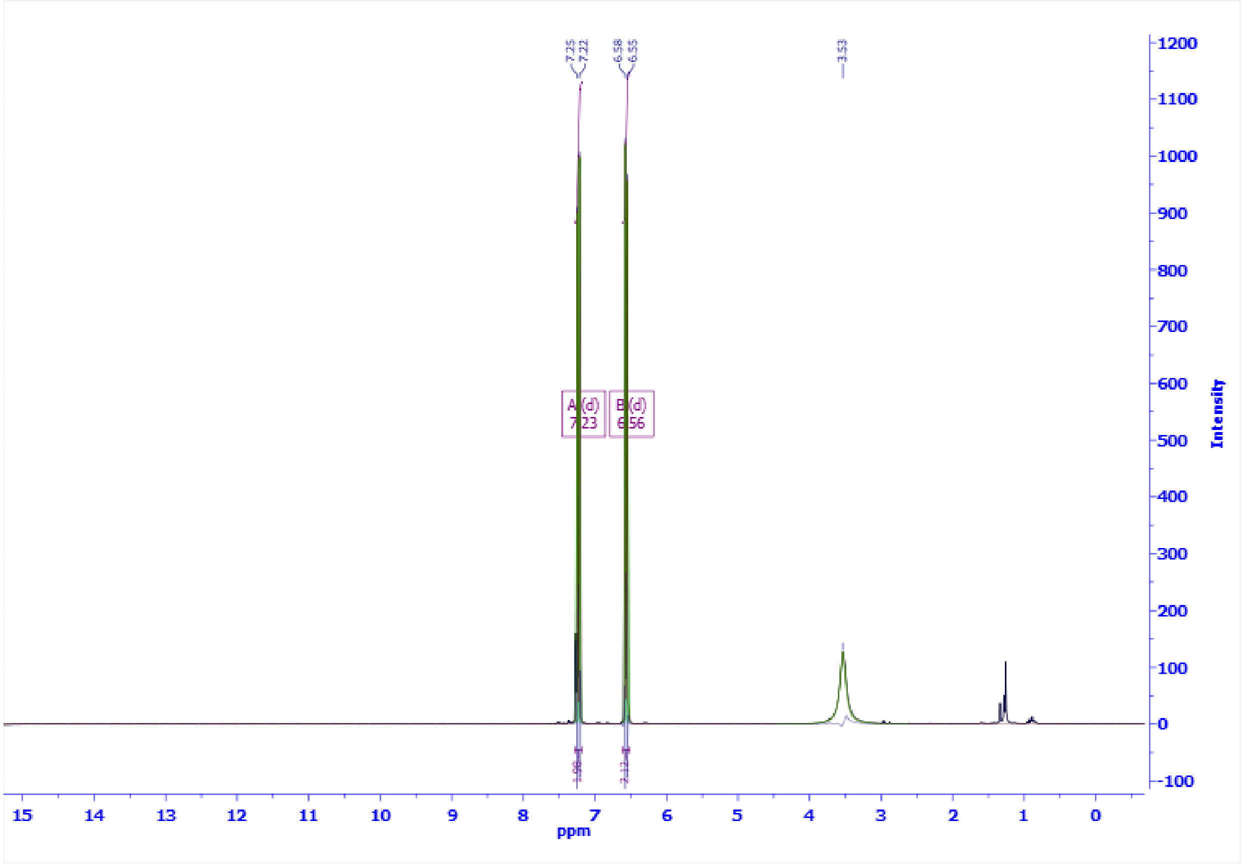

Figure 3. The ^{1}H NMR C

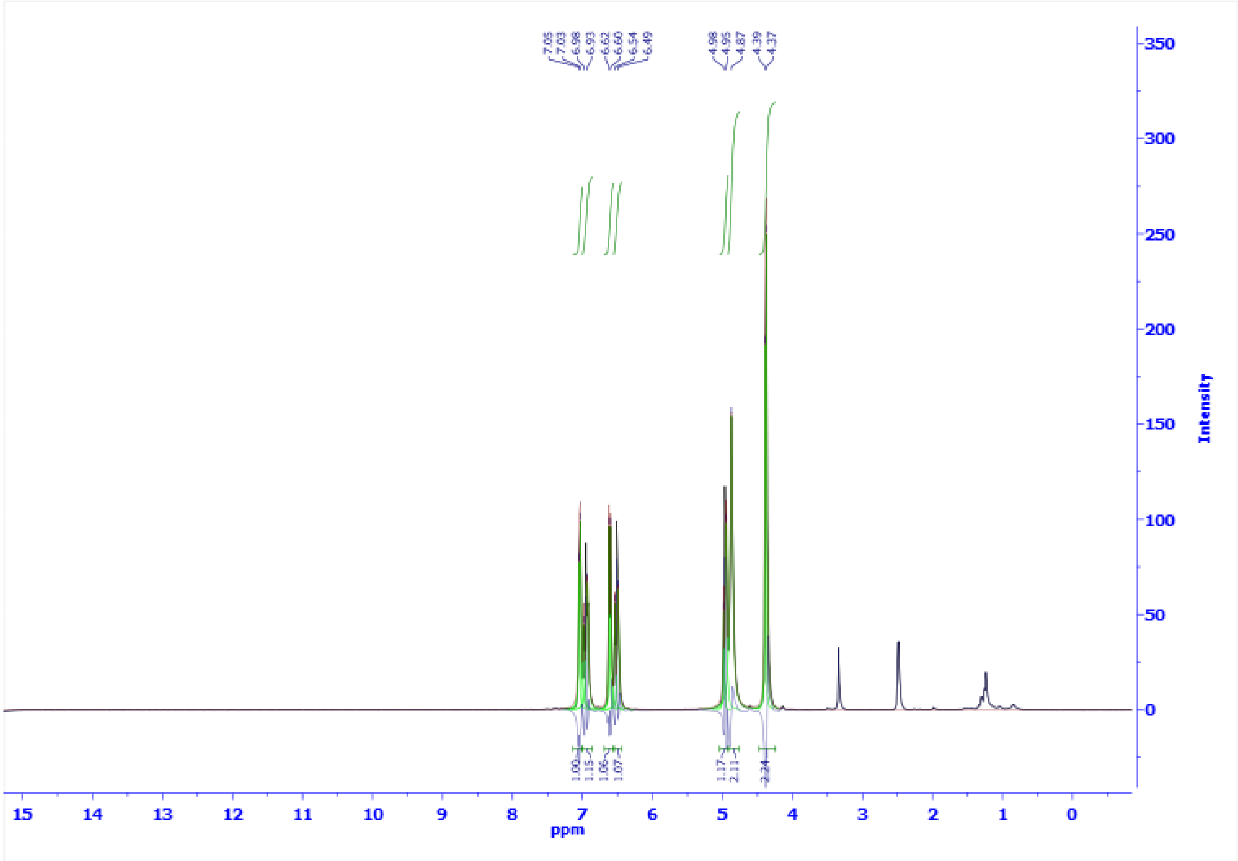

Figure 4. The ^{1}H NMR D
